# Efficient multi-allelic genome editing via CRISPR–Cas9 ribonucleoprotein-based delivery to *Brassica napus* mesophyll protoplasts

**DOI:** 10.3389/fpls.2024.1397632

**Published:** 2024-11-18

**Authors:** Sareena Sahab, Fatima Runa, Mahilini Ponnampalam, Pippa T. Kay, Elizabeth Jaya, Katerina Viduka, Stephen Panter, Josquin Tibbits, Matthew J. Hayden

**Affiliations:** ^1^ Agriculture Victoria, AgriBio, Centre for AgriBioscience, Melbourne, VIC, Australia; ^2^ School of Applied Systems Biology, La Trobe University, Melbourne, VIC, Australia

**Keywords:** canola, genome editing, CRISPR, Cas9, ribonucleoprotein, protoplasts

## Abstract

Canola (*Brassica napus* L.) is a valuable oilseed crop worldwide. However, trait improvement by breeding has been limited by its low genetic diversity and polyploid genetics. Whilst offering many potential benefits, the application of transgenic technology is challenged by the stringent and expensive regulatory processes associated with the commercialisation of genetically modified organisms, coupled with a prevailing low public acceptance of such modifications. DNA-free genome editing using Clustered Regularly Interspaced Short Palindromic Repeats (CRISPR)–Cas9 ribonucleoproteins (RNPs) offers a promising way to achieve trait improvements without the limitations of transgenic methods. Here, we present a method for DNA-free genome editing via the direct delivery of RNPs to canola mesophyll protoplasts. This method allows high-throughput *in vivo* testing of the efficacy of gRNA design as part of the transformation process to facilitate the selection of optimal designs prior to the generation of edited events. Of the 525 shoots regenerated via tissue culture from RNP-transfected protoplasts and screened for the presence of mutations in the targeted gene, 62% had one or more mutated target alleles, and 50% had biallelic mutations at both targeted loci. This high editing efficiency compares favourably with similar CRISPR–Cas9 approaches used in other crop plants.

## Introduction

Canola (*Brassica napus* L.) is a valuable oilseed crop with an annual global production value of 70–80 million tons in recent years and a total value of between 42 and 60 billion US dollars (Aaron and Maria, 2023). Canola is an allotetraploid species (AACC, 2n = 38) derived from a wide cross between *Brassica rapa* L. (AA, 2n = 20) and *Brassica oleracea* L. (CC, 2n = 18). Canola has low genetic diversity (Bus et al., 2011), and recent breeding for trait development has involved wide crosses, transgenic modifications, and fixing of spontaneous and induced mutations. Being amphidiploid complicates the induction of mutations with a measurable phenotype and often leads to an enormous background mutation load (Harloff et al., 2012). The probability of simultaneously inducing biallelic mutations that result in a phenotype is also extremely low, and redundancy caused by the presence of homeologous genes renders trait improvement by induced mutagenesis inefficient (Ali and Zhang, 2023). This can result in years of time-consuming crossing and backcrossing procedures to fix and deploy multiple mutations for trait improvement (Emrani et al., 2015).

Clustered Regularly Interspaced Short Palindromic Repeats (CRISPR)–Cas9 is a gene editing technology that involves two components: a guide RNA that matches the sequence of a target gene and a Cas9 (CRISPR-associated protein 9) endonuclease that introduces a double-stranded break into the targeted DNA sequence. Double-stranded breaks are subsequently repaired by an endogenous DNA repair mechanism, predominantly non-homologous end joining (NHEJ) in plants, which commonly results in small deletions within the targeted sequence owing to imprecise repair. CRISPR–Cas9 technology allows simultaneous editing of multiple homologues of target genes in polyploid plants, addressing the challenge of genetic redundancy (Lv et al., 2020; Li et al., 2024a, b). These attributes make the CRISPR–Cas9 technology attractive for trait improvement via highly targeted mutagenesis.

Cas9 can be delivered to cells as DNA, RNA, or preassembled ribonucleoproteins (RNPs) (Chen et al., 2021; Liu et al., 2022; Neelakandan et al., 2022; Bahariah et al., 2023; Zhou et al., 2023). RNPs have been successfully used for DNA-free genome editing in several plant species (Park and Choe, 2019) The key advantages of RNPs include the absence of exogenous DNA integration in genome-edited events in comparison to genome editing methods that involve plasmid DNA-based delivery and a reduced risk of off-target editing because RNPs are quickly degraded in plant cells (Ahmad et al., 2021). Despite these advantages of RNPs, a 2021 review of genome editing for trait development in canola reported that all applications of CRISPR–Cas9 technology at the time relied on plasmid DNA and *Agrobacterium*-mediated transformation (Gocal, 2021), and this method continues to be used (Hu et al., 2022). Li et al. (2021) reported the use of a CRISPR–Cas9 approach for editing the canola *GTR* and *GTR2* genes by direct delivery of plasmid DNA containing single guide RNA (sgRNA) and Cas9 expression cassettes. To the best of our knowledge, only Sidorov et al. (2021) have reported successful RNP-based gene editing in canola via polyethylene glycol (PEG)-mediated transfection of hypocotyl-derived protoplasts.

This study aimed to develop an efficient and robust exogenous DNA-free RNP-based delivery method for canola that is amenable to high-throughput genotypic screening and can be used for the rapid and routine generation of transgene-free edited events with modified agronomic traits. The main advantage of this method is its ability to generate highly targeted mutations in genes underlying key agronomic traits without the integration of exogenous DNA into the canola genome.

The 5′-untranslated region (UTR) of the Centromeric Histone H3 (*CENH3*) gene was chosen as the target sequence for our proof-of-concept experiments. *CENH3* is a specialised histone and an essential component of the kinetochore (Britt and Kuppu, 2016). Individual mutations and single-nucleotide polymorphisms in 5-UTRs have been reported to induce mutations, resulting in functional consequences (Diederichs et al., 2016; Schuster and Hsieh, 2019). The genomes of most diploid eukaryotic plants encode only one *CENH3* variant, whereas some species, including barley, rye, wheat, and cowpea, encode multiple variants (Sanei et al., 2011; Ishii et al., 2015; Evtushenko et al., 2017; Ishii et al., 2020; Evtushenko et al., 2021). In allotetraploid *Brassica* spp., one or both genes from the two ancestral subgenomes are co-expressed (Wang et al., 2011). Some partially functional *CENH3* coding region variants and point mutations are associated with haploid induction in a range of crops and could greatly accelerate commercial breeding programs (Ravi et al., 2011; Kelliher et al., 2019; Kuppu et al., 2020; Lv et al., 2020; Wang et al., 2021).

A range of methods, including Sanger or next-generation (NextGen) sequencing of amplicons, cleaved amplified polymorphic sequence (CAPS) assays, quantitative PCR, and digital PCR, have been used to detect mutations at loci targeted by CRISPR–Cas9 genome editing system plants (Liang et al., 2017; Andersson et al., 2018; Gao et al., 2018; Peng et al., 2020; Jouanin et al., 2020; Zhang et al., 2021; Shillito et al., 2021; Fraiture et al., 2022; Park et al., 2022; Rönspies et al., 2022; Anderson et al., 2023; Westberg et al., 2023; Wimberger et al., 2023; Lackner et al., 2023). In this study, a dual-probe droplet digital PCR (ddPCR) assay was employed as a rapid screen for the absolute quantification of target gene ablations, which were then validated using amplicon sequencing. This method was selected because of its exceptional sensitivity, precision, and accuracy. Additionally, it offers a cost-effective alternative to sequencing techniques and is well suited for high-throughput applications in polyploid plants, as demonstrated in studies by Gao et al. (2018); Jouanin et al. (2020), and Peng et al. (2020).

In this study, the use of a CRISPR–Cas9 system with commercially sourced RNP components generated mutations in the targeted canola gene with a high level of efficiency, and the results were comparable to those seen in a similar study in canola that utilised an RNP-based CRISPR–Cas12a system (Sidorov et al., 2021). In the current study, CRISPR–Cas9 RNP-based allelic mutations were successfully generated at the 5′-UTR region of the *BnCENH3* gene using Alt-R *S.p.* HiFi Cas9 Nuclease system (IDT Inc. USA) and along with custom sgRNA commercially sourced from IDT (IDT Inc., Newark, NJ, USA). The HiFi Cas9 Nuclease enzyme and custom sgRNA molecules, both sourced from Integrated DNA Technologies (Coralville, IA, USA), represent a substantial reduction in the cost of in-house lab work, with the advantage of using commercial reagents of high and consistent quality. Alt-R™ *S.p.* HiFi Cas9 Nuclease offers improved specificity over wild-type Cas9, significantly reducing the risk of off-target cleavage. This Cas9 variant also preserves the high level of editing efficiency expected from Cas9 nuclease, maintaining 90%–100% on-target editing activity at most sites (Vakulskas et al., 2018). A genetically modified organism (GMO)-free canola trait development pipeline that couples RNP-based genome editing based on commercially sourced reagents to a rapid, sensitive, precise, and efficient digital PCR screening method is likely to be of great value to canola breeding programs.

## Materials and methods

### Target site selection and spacer design

**Figure 1:**
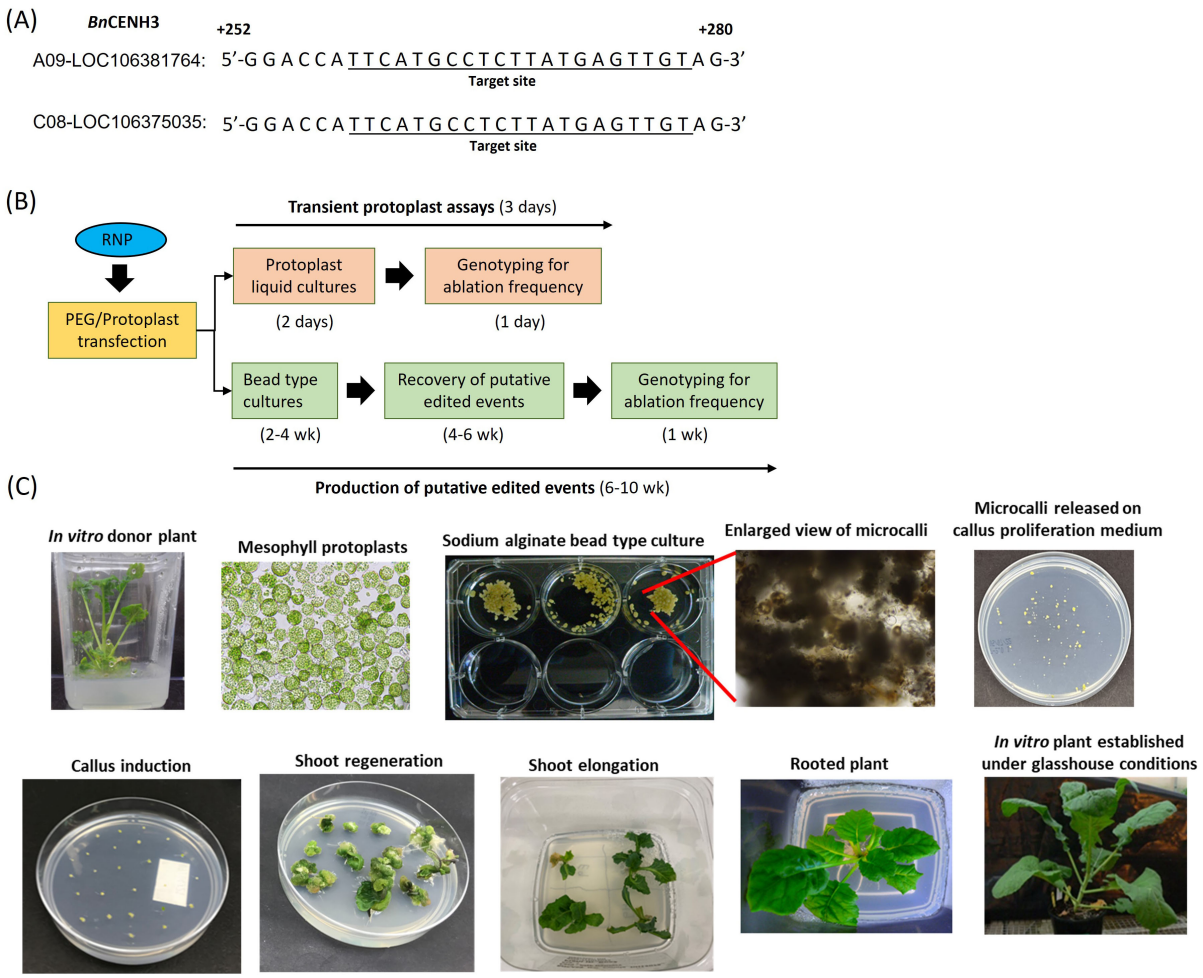
**(A)** Target sequences for Clustered Regularly Interspaced Short Palindromic Repeats (CRISPR)–Cas9 genome editing within the two *BnCENH3* homoeologs. The spacer sequence was designed using the genomic sequence of the DH12075 canola line. **(B)** Schematic diagram of the processes for transient assessment of gene editing efficiency and production of putative gene-edited events. In both processes, CRISPR–Cas9 ribonucleoprotein was directly delivered into mesophyll protoplasts using a polyethylene glycol (PEG)-mediated method. In transient assays, transfected protoplasts were recovered after 48 h and assessed for ablation frequency using a dual-probe droplet digital PCR (ddPCR) assay. To recover putative gene-edited events, protoplasts were regenerated through bead-type culture and tissue culture steps without selection. Leaf tissue samples from shoots were collected and screened for ablations using ddPCR. **(C)** Key steps involved in protoplast culturing in sodium alginate bead-type cultures and regeneration of canola plant.

A spacer sequence (**Supplementary Table 1**) was designed to recognise a 20-nucleotide site downstream of a Cas9 Protospacer Adjacent Motif (PAM) site (NGG) in the 5′-untranslated region of the *CENH3* (*BnCENH3*) gene (**Figure 1A**). The spacer sequence was designed using the genomic sequence of the *BnCENH3* gene from the canola line DH12075; the target sequences across the two *BnCENH3* homeologs (A09 and C08) are shown in **Figure 1A**. The spacer sequence was also checked to confirm that mutations at the predicted restriction site generated by the RNP complex could, in theory, be detected by a dual-probe ddPCR assay designed simultaneously. The spacer sequence was incorporated into a sgRNA (**Supplementary Table 1**), which was synthesised by Integrated DNA Technologies (IDT).

### Source of sgRNA and Cas9 protein

Custom Alt-R™ sgRNA and recombinant Alt-R *S.p.* HiFi Cas9 nuclease protein were purchased from IDT.

### Protoplast preparation and PEG-mediated delivery of CRISPR–Cas9 RNP

Mesophyll protoplasts were isolated from the DH12075 canola line using the method described by Sahab et al. (2019) with some modifications (**Supplementary Methods 1**). Critical factors determining the efficiency of each step are described in Sahab et al. (2019). CRISPR–Cas9 RNPs were prepared by combining 60 µg of Cas9 protein with 20 µg of sgRNA in 60 µL of transfection buffer or NE Buffer 3.1 (New England Biolabs, Inc., Ipswich, MA, USA). The transfection buffer contained 0.4 M mannitol, 15 mM MgCl_2_, and 4 mM morpholinoethanesulphonic acid (MES), pH 5.7. Aliquots of RNP were added to one million protoplasts, followed by 150 μL of PEG solution [polyethylene glycol (40% (w/v) PEG 4000 (source: Sigma, Darmstadt, Germany; Catalogue #81240) in 0.4 M mannitol, 0.1 M Ca (NO3)_2_; pH 6–7], and the samples were gently tapped to mix all the ingredients uniformly. After 15 min of incubation at room temperature, 10 mL wash buffer was added to the protoplast suspension. The protoplast suspension was gently mixed, and the protoplasts were pelleted by centrifugation using a swing-out rotor at 70 g for 10 min. The supernatant was removed, and the protoplast pellet was resuspended in 1 mL of fresh wash buffer (154 mM NaCl, 125 mM CaCl_2_, and 5 mM glucose; pH 5.8) (Menczel et al., 1981). Transfected protoplasts were incubated in a growth chamber for 48 h at 22°C with a 16-h photoperiod and an average light intensity of 300 μmol·m^−2^·s^−1^. In the case of transient studies, transfected protoplast pools were directly sampled for DNA extraction after 48 h of incubation. Transfected protoplasts were immediately embedded in a bead-type matrix in order to recover stably edited events (**Supplementary Methods 1**). A schematic representation of these processes is shown in **Figure 1B**. In brief, transfected protoplasts were embedded in sodium alginate bead-type cultures until visible microcalli were formed (~2–4 weeks). Initially, microcalli were placed on callus proliferation media. Subsequently, the surviving calli were transferred to shoot regeneration media to continue their development. This process facilitated the recovery of T0 plants with roots, which were transferred to a potting mix and cultivated under controlled glasshouse conditions (**Figure 1C**). The final recovery step, involving recovery of T0 plants from calli, took approximately 4–6 weeks. Overall, a 6–10-week time period was required to complete the recovery of all putative edited T0 events. Of the 1,200 T0 plants recovered, 525 were screened to identify edited events.

### DNA extraction from protoplasts and plants

Genomic DNA was extracted from protoplasts using the DNAdvance™ kit (Beckman Coulter, Inc., Brea, CA, USA), with minor modifications. Briefly, centrifugation at 10,000 × *g* for 4 min at 4°C was performed to concentrate protoplast samples, and the supernatant was removed. Each pellet was resuspended in 100 µL DNAdvance™ Lysis Master Mix, vortex-mixed, and incubated for 15 min at 55°C. Samples were cooled on ice for 2 min, mixed with 50 µL of pre-chilled 7.5 M ammonium acetate, and incubated for 1 hour on ice. Following centrifugation at 10,000 × *g* for 10 min at 4°C, 100 µL of the supernatant from each sample was aspirated and combined with 50 µL of DNAdvance™ Bind 1 solution, vortex-mixed, and combined with 85 µL of DNAdvance™ Bind 2 solution. After vortexing, the samples were incubated for 5 min at ambient temperature and placed on a magnet for 4 min to separate the beads. The supernatant was discarded, and the beads were rinsed three times with 200 µL of 70% v/v ethanol before brief air-drying on the magnet. Genomic DNA was eluted in 30 µL of DNAdvance™ Elution Buffer.

Samples of immature leaf tissue (~100 mg) were collected from T0 plants grown in the glasshouse and placed in racked collection microtubes (Qiagen, Valencia, CA, USA). Leaf tissue was freeze-dried for 48 h prior to DNA extraction. Genomic DNA was isolated using the DNeasy™ 96 Plant Kit (Qiagen), according to the manufacturer’s protocol. Purified DNA samples were quantified using a NanoDrop™ 2000 spectrophotometer (Thermo Fisher Scientific Inc., Waltham, MA, USA) and diluted to 5 ng/μL for genotyping using a ddPCR assay.

### Estimation of *BnCENH3* ablation efficiency by droplet digital PCR and sequence characterisation of gene edits

**Figure 2:**
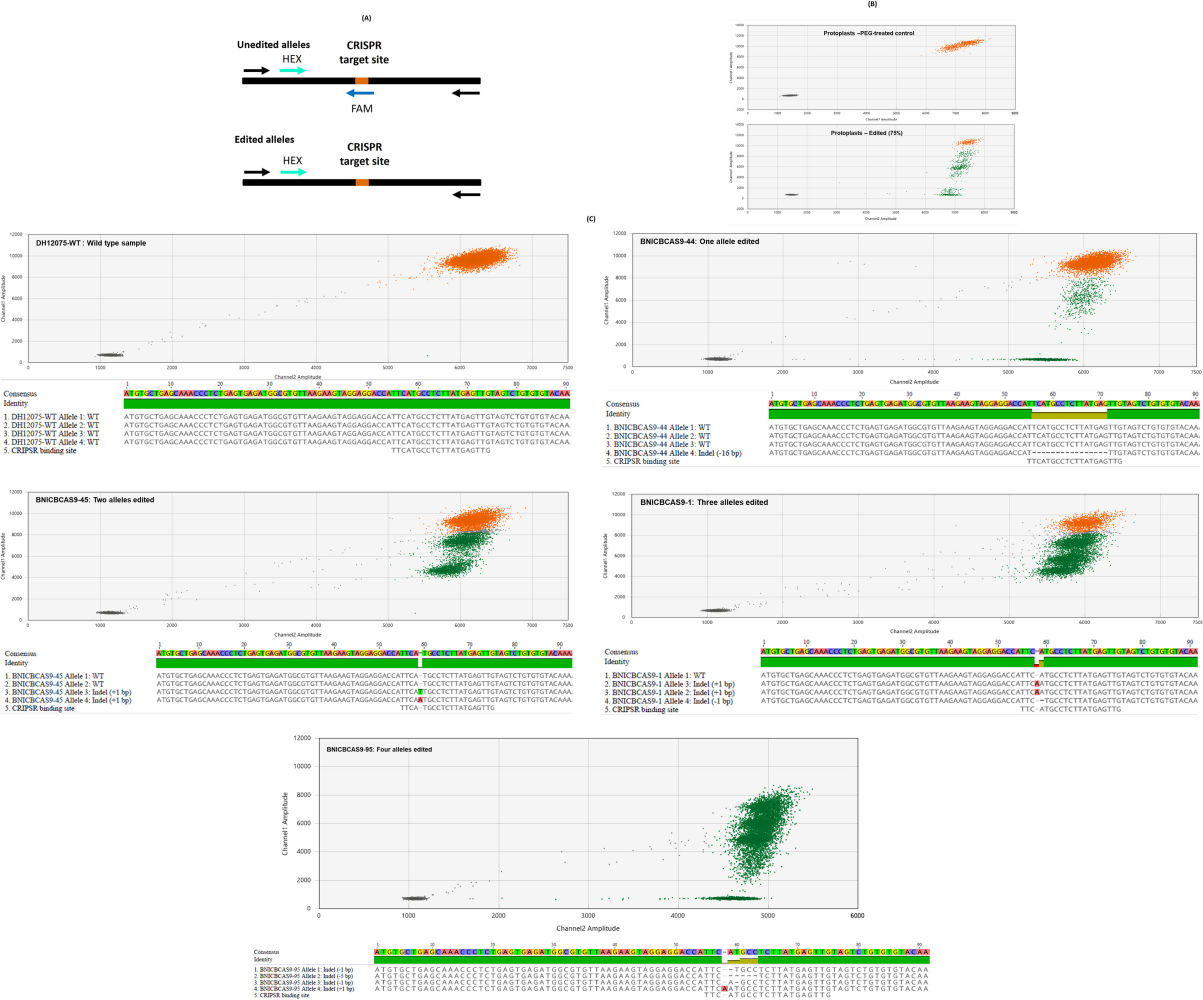
**(A)** Schematic diagram of the single-amplicon dual-probe droplet digital PCR (ddPCR) assay used to assess ablation. Black and coloured arrows indicate the locations of the primers and probes, respectively. Probes labelled at the 5′ end with FAM and HEX fluorophores were designed to span the Clustered Regularly Interspaced Short Palindromic Repeats (CRISPR) target site and a different site within the target gene, respectively. Both probes bind to the wild-type allele of the target gene, resulting in both HEX and FAM fluorescence (unedited allele). Only the HEX probe binds to a mutated allele of the target gene because of the disruption of the FAM probe binding site caused by CRISPR–Cas9 activity (edited allele). **(B)** Representative ddPCR droplet plots for protoplast samples. **(C)** Representative ddPCR droplet plots for assays performed using genomic DNA (top panel; the droplet amplitudes on the x- and y-axes correspond to HEX and FAM fluorescence, respectively) extracted from T0 canola plants and their corresponding *CENH3* allele sequences (bottom panel; FASTA reads of the wild-type and gene-edited alleles for each plant were aligned and visualised using Geneious version 2024.0 created by Biomatters, available from https://www.geneious.com). Droplets representing the wild-type and mutated alleles of the target gene are shown in orange and green, respectively. Edited plants were predicted to have one, two, three, and four mutated alleles, respectively, based on non-wild-type droplet frequencies of approximately 25%, 50%, 75%, and 100%.

A dual-probe ddPCR assay, also called a drop-off or ablation assay, was used to measure ablation efficiency (**Figure 2A**). The assay was designed using Sequencher 5.0 (Gene Codes, Ann Arbor, MI, USA) and the IDT PrimerQuest™ tool. Primer and probe details are shown in **Supplementary Table 2**. A FAM-labelled probe spanning the predicted CRISPR–Cas9 ablation site in the *CENH3* gene and a HEX-labelled probe matching another part of *CENH3* were located within an amplicon flanked by a forward primer and a reverse primer. ddPCR assays were performed in a 24-µL reaction volume in Twin Tec™ 96-well microplates (Eppendorf) comprising 1× ddPCR Master Mix (no UTP, Bio-Rad Laboratories, Hercules, CA, USA), 0.25 µM HEX- and FAM-labelled probes (IDT), 0.5 µM forward and reverse primers (IDT), and 10 ng of genomic DNA. Reactions were prepared for PCR using an automated droplet generator (Bio-Rad Laboratories), sealed with pierceable foil heat seals (Bio-Rad Laboratories), and cycled using C1000 Touch™ thermocyclers (Bio-Rad Laboratories) with an initial 95°C denaturation for 10 min, followed by 39 cycles of denaturation at 95°C for 30 s, annealing at 59°C for 30 s, and extension at 72°C for 2 min, followed by a final extension at 72°C for 5 min and denaturation at 95°C for 10 min. A ramp rate of 2°C/sec was used for all the steps. Data were collected using a QX200™ Droplet Reader, and droplet clusters were analysed using QuantaSoft™ Analysis Pro software (Bio-Rad Laboratories).

Representative plants from each of the observed ddPCR clustering patterns identified from the assessment of the T0 events were characterised by amplicon sequencing. A detailed description of the approach used for amplicon library preparation, sequencing, and data analysis is provided in **Supplementary Methods 2**.

## Results

### Assessment of *BnCENH3* ablation efficiency in canola protoplasts

PEG-mediated transfection of mesophyll protoplasts with CRISPR–Cas9 RNPs was performed with either New England Biolabs Buffer 3.1 or transfection buffer. Protoplast transfections without RNPs were used as a negative control. Experiments involving RNPs were performed with two biological replicates derived from independent protoplast preparations and at least three technical replicates (**Table 1A**). Analysis of genomic DNA from mesophyll protoplasts transfected with RNPs showed evidence of CRISPR–Cas9-mediated *CENH3* allele editing (**Figure 2B**). As expected, the PEG-only negative control assay showed a large cluster of droplets with high amplitudes of both HEX and FAM fluorescence (orange data points), corresponding to wild-type *CENH3* alleles. Assays that involved CRISPR–Cas9 RNP delivery showed smaller clusters of droplets having a high amplitude of HEX fluorescence but reduced FAM fluorescence (green data points), corresponding to *CENH3* alleles that were mutated at the target site by CRISPR–Cas9 RNP activity (**Figure 2B**). The frequency of mutated *CENH3* alleles ranged from 65% to 76% in the genomic DNA derived from protoplasts treated with RNPs (**Table 1A**; **Supplementary Table 3**). A slightly lower mutation frequency was observed when the RNPs were delivered in the transfection buffer than in NEB Buffer 3.1. Despite this, transfection buffer was used for RNP deliveries aimed at regenerating edited canola plants from protoplasts because of its established use in transformation and regeneration processes (Sahab et al., 2019).

**Table 1A:** Estimated frequencies of mutated *CENH3* alleles in mesophyll protoplasts after CRISPR–Cas9 RNP delivery.

Treatment	Biological replicate number	Number of technical replicates	Ablation frequency (%) Mean ± SD
1	1	4	76 ± 1.6
1	2	3	75 ± 1.0
2	1	4	69 ± 0.6
2	2	4	65 ± 0.2
3	1	4	0 ± 0.2

In treatments 1 and 2, RNPs were delivered in Buffer 3.1 (New England Biolabs) and Transfection Buffer, respectively. Treatment 3 was the PEG-only negative control. Ablations were detected using a droplet digital PCR assay and analysed using QuantaSoft™ analysis Pro Software (Bio-Rad).

CRISPR, Clustered Regularly Interspaced Short Palindromic Repeats; RNP, ribonucleoprotein; PEG, polyethylene glycol.

### Recovery of canola plants with *BnCENH3* mutations

Approximately 1,200 protoplast-derived T0 plants were regenerated from mesophyll protoplasts in tissue culture following transfection with CRISPR–Cas9 RNPs, of which 525 were tested for gene ablations. The high recovery of T0 plants was due to the utilisation of an established protoplast transformation and plant regeneration method (Sahab et al., 2019) in which critical factors determining the success of each key step had been resolved. Genotyping of individual plants by ddPCR showed that they had droplet frequencies corresponding to non-wild-type versions of the canola *CENH3* gene between 0% and 100% (**Table 1B**; **Supplementary Table 4**). The DH12075 canola line used in this study is known to have two copies of the *CENH3* gene, located on chromosomes A09 and C08, both of which were targeted by CRISPR–Cas9 RNPs. Therefore, on a per-plant basis, non-wild-type droplet frequencies of 0%, 25%, 50%, 75%, and 100% were expected to correspond to the editing of 0, 1, 2, 3, and 4 *CENH3* alleles, respectively (**Figure 2C**). Overall, 68.2% of the plants were edited at the *CENH3* loci, with 4.8%, 5.7%, 8.2%, and 50% having one, two, three, and four mutated *CENH3* alleles, respectively. Amplicon-based sequence characterisation of representative samples revealed corresponding allelic mutations consistent with the ddPCR frequencies (**Figure 2C**). Based on the sequencing data, only one type of allele was observed in the unedited (wild-type) samples where the CRISPR binding site was intact, and the amplicon size was as expected (171 bp long). In contrast, the T0 plants had between one and four mutated alleles with small insertions and deletions (**Figure 2C**). The number of mutated alleles per T0 plant concurred with the observed percentage of ablation droplets for that plant in the ddPCR analysis. The observation that half of the plants had four mutated alleles indicated that the biallelic editing of both endogenous *CENH3* loci was highly efficient.

**Table 1B:** Estimated number of mutated *CENH3* alleles in canola plants after delivery of CRISPR–Cas9 RNPs.

Estimated number of mutated alleles	Number of tested plants	Percentage of tested plants
0	167	31.8
1	25	4.8
2	30	5.7
3	43	8.2
4	260	49.5
	525	100

Genomic DNA was extracted from shoots regenerated from protoplasts transfected with RNPs and analysed using a droplet digital PCR assay. The number of mutated alleles was estimated based on the percentage of non-wild-type droplets.

CRISPR, Clustered Regularly Interspaced Short Palindromic Repeats; RNP, ribonucleoprotein.

## Discussion

Genome editing has the potential to create targeted mutations in the genes of crop plants that underlie key agronomic traits, resulting in modified gene expression or protein function. DNA-free genome editing approaches do not involve the delivery of exogenous DNA and are expected to have a lower regulatory burden than DNA-based editing methods and transgenesis. Despite its importance as an oilseed crop, to our knowledge, only one successful DNA-free genome editing approach for canola has been reported (Sidorov et al., 2021). This study demonstrated that a CRISPR–Cas9 RNP delivery method utilising commercially available RNP components coupled with a ddPCR screening method represents a highly efficient DNA-free genome editing method in canola with potential value for canola trait development.

### Development of rapid screening methods to assess genome editing efficiency

Screening of genomic DNA from protoplasts transfected with CRISPR–Cas9 RNPs by ddPCR represents a robust and rapid *in vivo* method for testing the efficacy of sgRNA designs. Whilst rapid *in vitro* tests for assessing the efficacy of gRNA designs using CRISPR–Cas9 RNPs are commercially available to validate cut site specificity, they do not accurately predict *in vivo* performance because they are not as quantitative as ddPCR, and short target molecules of DNA lack the DNA methylation patterns of genomic DNA in living cells, which is known to inhibit Cas9 activity, if present (Přibylová et al., 2022). These limitations can be overcome with the use of a ddPCR-coupled protoplast assay that allows RNP activity to be tested on targets within the genomic DNA inside plant cells. Overall, this rapid screening method, combining protoplast transfection and a dual-probe ddPCR-based ablation assay, has the potential to test large numbers of sgRNA designs against multiple targets at a relatively low cost, provided that the protoplast system is amenable to high-throughput microplate-based transfection and recovery of a sufficient quantity of genomic DNA for genotyping.

### High-frequency editing of *BnCENH3* homeologs using CRISPR–Cas9 RNP delivery

When protoplasts were transfected with CRISPR–Cas9 RNPs, mutation frequencies of between 65% and 76% in *CENH3* alleles were measured. Plants regenerated from transfected protoplasts had mutation frequencies of approximately 68% in *CENH3* alleles. Of the 525 T0 events tested, approximately 50% had biallelic mutations at both *CENH3* loci, i.e., mutations in all four *CENH3* alleles (**Tables 1A**, **B**). These results compare favourably with CRISPR–Cas9 RNP delivery experiments in a range of crop plants (reviewed by Metje-Sprink et al., 2019). Murovec et al. (2018) achieved mutation rates of between 2.5% and 24.5% in *B. oleracea* and *B. rapa* protoplasts following the delivery of CRISPR–Cas9 RNPs targeting the phytoene desaturase gene and homologues of the *FRIGIDA* gene. Sidorov et al. (2021) reported a biallelic editing frequency of up to 50% using RNPs containing LbCas12a and a sgRNA targeting an intergenic region in *B. napus* protoplasts and regenerated plants. Our results support those of Sidorov et al. (2021) in demonstrating that a CRISPR–Cas9 or CRISPR–Cas12a RNP system involving direct delivery of RNPs to protoplasts can be used for efficient DNA-free genome editing in canola. We also showed that DNA extracted from transfected protoplasts and regenerated plants can be screened for editing efficiency rapidly and in high throughput using the robust and established digital PCR method (**Figure 2C**).

### Generation of transgene-free plants with targeted mutations for agronomic trait development

A CRISPR–Cas9 RNP approach to generate transgene-free plants with targeted mutations is likely to become a valuable tool to augment conventional breeding and germplasm development, with a lower regulatory burden than DNA-based genome editing methods. There is also no need for in-house vector construction, characterisation of exogenous DNA in edited plants, and its removal by segregation. The major impediments in generating transgene-free edited plants using direct RNP delivery have been the efficient delivery of RNPs into cells and the selection and recovery of edited plants in the absence of a selectable/screenable marker. In this study, we demonstrated that the effective delivery of CRISPR–Cas9 RNPs into cells can be achieved using a PEG-based protoplast transfection system in canola. Efficient RNP delivery leading to a high rate of cellular uptake appears to result in a high genome editing rate, presumably due to the efficacy of the RNP complex. The high editing efficiency, demonstrated by 62% of the recovered plants carrying mutations in at least one of the targeted *CENH3* alleles, shows that the use of a selectable marker is not needed for the recovery of a large number of edited plants. Our study suggests that edited canola plants with a desired allelic profile can be readily recovered, making this system a robust method for the creation and recovery of canola plants with targeted biallelic mutations in multiple homeologs of multicopy genes using a completely DNA-free system.

In conclusion, we have demonstrated an efficient DNA-free approach for efficiently generating gene-edited canola germplasm, as well as a high-throughput *in vivo* screening assay for assessing the efficacy of new CRISPR gRNA designs. Further work will focus on the comprehensive sequence-based analysis of edited events, the development of multiplexing strategies, and the testing of elite canola germplasm to facilitate the deployment of this technology into breeding systems.

## Data Availability

The original contributions presented in the study are included in the article/**Supplementary Material**. Further inquiries can be directed to the corresponding author.

## Supplementary material

Supplementary Methods 1 Protoplast isolation and recovery of edited events.

Supplementary Methods 2 Amplicon sequencing method and data analysis.

Supplementary Table 2 ddPCR probes and primers used to quantify the frequency of mutations in *CENH3* alleles.

Supplementary Table 3 Summary table of droplet digital PCR ablation assay for canola protoplast samples transfected with a CRISPR-CAS9 RNPs targeting the *CENH3* gene.

Supplementary Table 4 Raw results of droplet digital PCR ablation assay for canola shoot samples derived from protoplasts transfected with a CRISPR-CAS9 RNP targeting *BnCENH3* homoeologs.
