# Supplementary material for: Efficient multi-allelic genome editing via CRISPR–Cas9 ribonucleoprotein-based delivery to Brassica napus mesophyll protoplasts
Source: Front Plant Sci. 2024 Nov 18;15:1397632. doi: 10.3389/fpls.2024.1397632 (PMC11608969; doi:10.3389/fpls.2024.1397632)
Supplement: Supplementary Methods 1 — Protoplast isolation and recovery of edited events. [file DataSheet4.pdf]

Amplicon library preparation

A total of 16 plants were selected for amplicon sequencing. These included three plants from each of the five genotypic groups observed from droplet-digital PCR: wild type, single edited allele, two edited alleles, three edited alleles or four edited alleles of the canola *CENH3* gene. A single DH12075 wild type plant that had not been through the genome editing process was used as a negative control. Genomic DNA was extracted from leaf tissue samples using the MagMAX™ Plant DNA Isolation Kit and the Kingfisher Flex™ automated extraction system (Thermo Fisher Scientific) according to the manufacturer's protocol. Genomic DNA was quantified using a Nanodrop™ 8000 spectrophotometer (Thermo Fisher Scientific) and diluted to 25 ng/µL. Approximately 50 ng of plant genomic DNA was used in each 50 µL primary PCR reaction. Gene-specific primers, manufactured by Integrated DNA Technologies, were tailed to allow the use of paired-end (PE) sequencing technology. Details of PCR components and cycling conditions are shown in the tables below.

Sequences of PCR Primers and CRISPR Binding Site Within Canola CENH3 Genes

Primer Name	Sequence
Bn_R026_R035_F072_f2_NGS	ACA CTC TTT CCC TAC ACG ACG CTC TTC CGA TCT ATG TGC TGA GCA AAC CCT CT
Bn_R026_R035_F072_r1_NGS	TGA CTG GAG TTC AGA CGT GTG CTC TTC CGA TCT AGA TTA TTT GTT TTC CCG CTT CTC C
R026 CRISPR binding site	TTCATGCCTCTTATGAGTTG

**Name**	**Gene-Specific Sequences (5'-3')**	**PE Tail Sequences (5'-3')**
Forward primer	ATGTGCTGAGCAAACCCTCT	ACACTCTTTCCCTACACGACGCTCTTCC GATCT
Reverse primer	GGAGAAGCGGGAAAACAAATAATCT	TGACTGGAGTTCAGACGTGTGCTCTTCC GATCT
R026 CRISPR binding site	TTCATGCCTCTTATGAGTTG	

Primary PCR Components

**Component Details**	**Volume (µL)**
Ultrapure™ DNAse/RNAse-Free Distilled Water (Thermo Fisher Scientific)	19
KAPA™ HiFi HotStart ReadyMix, 2x (Roche)	25
SYBR Green I Nucleic Acid Gel Stain, diluted 1000x (Thermo Fisher Scientific)	1
Forward primer (10 µM)	1.5
Reverse primer (10 µM)	1.5
Plant genomic DNA (25 ng/uL)	2
Total volume	50

hermal Cycling Conditions for Primary PCR

Step	Details	Temperature (°C)	Time (sec)
1	Initial denaturation	95	180
2	Denaturation	98	20
3	Annealing	60	15
4	Extension	72	15
5	Repeat steps 2-4, 25-34x	n/a	n/a
6	Hold	4	n/a

Amplification was performed using a CFX Touch™ real-time thermocycler with Quantasoft™ software (Bio-Rad Laboratories). Cycling was stopped when amplification was approximately 75% complete, according to SYBR™ Green fluorescence measurements. This corresponded to the 29th cycle. Amplicons were purified with Ampure XP™ SPRI (Solid Phase Reversible Immobilisation) beads (Beckman-Coulter Life Sciences) using a double purification method involving 0.6 and 0.9 bead-to-sample ratios in sequential steps. Purified amplicons were quantified using a Nanodrop™ 2000 spectrophotometer (Thermo Fisher Scientific) and diluted to approximately 0.5 ng/µL to use as templates for secondary PCR, a multiplexing PCR step where dual-indexed barcodes and sequencing flowcell-compatible sequences were attached to the amplicon library. Up to 1 ng of purified amplicon DNA from the primary PCR step was used as the template for secondary PCR reactions, which were performed using real-time PCR amplification in the same way as the primary PCR reaction. Cycling was stopped when amplification was approximately 75% complete, according to SYBR™ Green fluorescence. This corresponded to the 12th cycle. Details related to secondary PCR reactions are shown in the tables below.

Secondary PCR Components

Component Details	Volume (µL)
Ultrapure™ DNAse/RNAse-Free Distilled Water (Thermo Fisher Scientific)	20
KAPA™ HiFi HotStart ReadyMix, 2x (Roche)	25
SYBR Green I Nucleic Acid Gel Stain, diluted 1000x (Thermo Fisher Scientific)	1
B-mpx equimolar_PE1/PE2 primer mixture (2.5 µM)	2
Purified primary PCR amplicon (0.5 ng/µL)	2
Total volume	50

Sequences of Secondary PCR Primers

Sample Name	PE Index ID	Index	Sequence
DH12075WT	mpxPE2.bc008	AGGACAGT	C*AA GCA GAA GAC GGC ATA CGA GAT AAC TGT CCT GTG ACT GGA GTT CAG ACG TGT
BNICBCAS9_43	mpxPE2.bc004	CATTAACG	C*AA GCA GAA GAC GGC ATA CGA GAT GCG TTA ATG GTG ACT GGA GTT CAG ACG TGT
BNICBCAS9_50	mpxPE2.bc004	CATTAACG	C*AA GCA GAA GAC GGC ATA CGA GAT GCG TTA ATG GTG ACT GGA GTT CAG ACG TGT
BNICBCAS9_36	mpxPE2.bc003	TAGTGGCA	C*AA GCA GAA GAC GGC ATA CGA GAT TTG CCA CTA GTG ACT GGA GTT CAG ACG TGT
BNICBCAS9_25	mpxPE2.bc003	TAGTGGCA	C*AA GCA GAA GAC GGC ATA CGA GAT TTG CCA CTA GTG ACT GGA GTT CAG ACG TGT
BNICBCAS9_44	mpxPE2.bc004	CATTAACG	C*AA GCA GAA GAC GGC ATA CGA GAT GCG TTA ATG GTG ACT GGA GTT CAG ACG TGT
BNICBCAS9_34	mpxPE2.bc003	TAGTGGCA	C*AA GCA GAA GAC GGC ATA CGA GAT TTG CCA CTA GTG ACT GGA GTT CAG ACG TGT
BNICBCAS9_15	mpxPE2.bc002	GACGAGAT	C*AA GCA GAA GAC GGC ATA CGA GAT AAT CTC GTC GTG ACT GGA GTT CAG ACG TGT
BNICBCAS9_45	mpxPE2.bc004	CATTAACG	C*AA GCA GAA GAC GGC ATA CGA GAT GCG TTA ATG GTG ACT GGA GTT CAG ACG TGT
BNICBCAS9_6	mpxPE2.bc001	TACGAAGT	C*AA GCA GAA GAC GGC ATA CGA GAT GAC TTC GTA GTG ACT GGA GTT CAG ACG TGT
BNICBCAS9_1	mpxPE2.bc001	TACGAAGT	C*AA GCA GAA GAC GGC ATA CGA GAT GAC TTC GTA GTG ACT GGA GTT CAG ACG TGT
BNICBCAS9_113	mpxPE2.bc006	TAGTACGC	C*AA GCA GAA GAC GGC ATA CGA GAT AGC GTA CTA GTG ACT GGA GTT CAG ACG TGT
BNICBCAS9_115	mpxPE2.bc006	TAGTACGC	C*AA GCA GAA GAC GGC ATA CGA GAT AGC GTA CTA GTG ACT GGA GTT CAG ACG TGT
BNICBCAS9_11	mpxPE2.bc001	TACGAAGT	C*AA GCA GAA GAC GGC ATA CGA GAT GAC TTC GTA GTG ACT GGA GTT CAG ACG TGT
BNICBCAS9_2	mpxPE2.bc001	TACGAAGT	C*AA GCA GAA GAC GGC ATA CGA GAT GAC TTC GTA GTG ACT GGA GTT CAG ACG TGT
BNICBCAS9_95	mpxPE2.bc005	TCGTTGAA	C*AA GCA GAA GAC GGC ATA CGA GAT CTT CAA CGA GTG ACT GGA GTT CAG ACG TGT
DH12075WT	mpxPE1.bc025	TGTAACGT	A*AT GAT ACG GCG ACC ACC GAG ATC TAC ACT GTA ACG TCA CAC TCT TTC CCT ACA CGA C
BNICBCAS9_43	mpxPE1.bc017	AGGTAGAC	A*AT GAT ACG GCG ACC ACC GAG ATC TAC ACA GGT AGA CTA CAC TCT TTC CCT ACA CGA C
BNICBCAS9_50	mpxPE1.bc021	ACCAGTTG	A*AT GAT ACG GCG ACC ACC GAG ATC TAC ACA CCA GTT GAA CAC TCT TTC CCT ACA CGA C
BNICBCAS9_36	mpxPE1.bc022	GAGATCGA	A*AT GAT ACG GCG ACC ACC GAG ATC TAC ACG AGA TCG ATA CAC TCT TTC CCT ACA CGA C
BNICBCAS9_25	mpxPE1.bc017	AGGTAGAC	A*AT GAT ACG GCG ACC ACC GAG ATC TAC ACA GGT AGA CTA CAC TCT TTC CCT ACA CGA C
BNICBCAS9_44	mpxPE1.bc018	ACTACCGA	A*AT GAT ACG GCG ACC ACC GAG ATC TAC ACA CTA CCG ATA CAC TCT TTC CCT ACA CGA C
BNICBCAS9_34	mpxPE1.bc021	ACCAGTTG	A*AT GAT ACG GCG ACC ACC GAG ATC TAC ACA CCA GTT GAA CAC TCT TTC CCT ACA CGA C
BNICBCAS9_15	mpxPE1.bc018	ACTACCGA	A*AT GAT ACG GCG ACC ACC GAG ATC TAC ACA CTA CCG ATA CAC TCT TTC CCT ACA CGA C
BNICBCAS9_45	mpxPE1.bc019	CATCATAC	A*AT GAT ACG GCG ACC ACC GAG ATC TAC ACC ATC ATA CGA CAC TCT TTC CCT ACA CGA C
BNICBCAS9_6	mpxPE1.bc020	TTACGACA	A*AT GAT ACG GCG ACC ACC GAG ATC TAC ACT TAC GAC AGA CAC TCT TTC CCT ACA CGA C
BNICBCAS9_1	mpxPE1.bc017	AGGTAGAC	A*AT GAT ACG GCG ACC ACC GAG ATC TAC ACA GGT AGA CTA CAC TCT TTC CCT ACA CGA C
BNICBCAS9_113	mpxPE1.bc018	ACTACCGA	A*AT GAT ACG GCG ACC ACC GAG ATC TAC ACA CTA CCG ATA CAC TCT TTC CCT ACA CGA C
BNICBCAS9_115	mpxPE1.bc019	CATCATAC	A*AT GAT ACG GCG ACC ACC GAG ATC TAC ACC ATC ATA CGA CAC TCT TTC CCT ACA CGA C
BNICBCAS9_11	mpxPE1.bc023	CATAGTTG	A*AT GAT ACG GCG ACC ACC GAG ATC TAC ACC ATA GTT GCA CAC TCT TTC CCT ACA CGA C
BNICBCAS9_2	mpxPE1.bc018	ACTACCGA	A*AT GAT ACG GCG ACC ACC GAG ATC TAC ACA CTA CCG ATA CAC TCT TTC CCT ACA CGA C
BNICBCAS9_95	mpxPE1.bc018	ACTACCGA	A*AT GAT ACG GCG ACC ACC GAG ATC TAC ACA CTA CCG ATA CAC TCT TTC CCT ACA CGA C

*phosphorothioate linkages

Thermal Cycling Conditions for Secondary PCR

Step	Details	Temperature (ºC)	Time (sec)
1	Initial denaturation	95	180
2	Denaturation	98	20
3	Annealing	65	15
4	Extension	72	15
5	Repeat steps 2-4, up to 20 times	n/a	n/a
6	Hold	4	n/a

Amplicons from the 16 plants were pooled. The quantity of each reaction added to the pool was normalised using endpoint fluorescence readings from the individual PCR reactions. The pooled library was purified using Ampure XP™ beads (Beckman-Coulter) using a 0.7x bead-to-sample ratio. The average molecular size of the library and its DNA concentration were determined using a TapeStation™ 4200 instrument (Agilent), a Qubit™ fluorometer (Thermo Fisher Scientific) and a KAPA™ Library Quantification Kit (Roche), according to the manufacturer's protocols. The MiSeq™ Sequencing System (Illumina) with the MiSeq™ Reagent Nano Kit v2 (300 cycle) were used to sequence the amplicon library. The amplicon size was determined to be 171 bp, excluding barcodes and PE tails used to facilitate sequencing. The CRISPR binding site was located between 55-74 bp from the forward PCR primer in the 5'-3' strand of the amplicon. The library pool was sequenced to obtain 220 bp and 80 bp of sequence data from Read 1 and Read 2, respectively. Read 1 covered most of the amplicon sequence, including the CRISPR binding site. Read 2 covered the opposite end of the amplicon.

Analysis of paired-end sequence data

PEAR 0.9.8 software (https://cme.h-its.org/exelixis/web/software/pear/) was used to merge Read 1 and Read 2 data into single sequences. An in-house PERL script was used to find unique sequences and count their frequencies. Sample names were assigned to the unique sequences, along with their frequencies, and combined in a single FASTA file. The FASTA file was imported into Microsoft Excel™ and added to a table containing results from the droplet-digital PCR analysis. The 20 bp sequence of the CRISPR-Cas9 binding site was used to scan the list of unique sequences to identify wildtype and edited alleles. Unique sequences with an insignificant frequency relative to the total number of reads generated from a sample were excluded from the analysis. The total number of wild type and gene-edited reads was calculated by summing the frequency count of each unique read. Gene-edited reads that contained different mutations were considered to represent different *CENH3* alleles. Finally, the proportion of wild type and gene-edited alleles was checked to confirm they had expected representation as a percentage of the total read count for the sample. For example, a wild type plant was expected to have 100% wild type reads, whereas plants with one gene edited allele were expected to have about 75% wild type reads and about 25% gene edited reads. Similarly, plants with two gene edited alleles were expected to have about 50% wild type reads, about

25% of reads corresponding to the first gene edited allele and about 25% reads corresponding to the second gene edited allele. FASTA reads of the wild type and gene-edited alleles for each plant were aligned and visualised using Geneious version 2024.0 created by Biomatters. Available from https://www.geneious.com.
