# Supplementary material for: Efficient multi-allelic genome editing via CRISPR–Cas9 ribonucleoprotein-based delivery to Brassica napus mesophyll protoplasts
Source: Front Plant Sci. 2024 Nov 18;15:1397632. doi: 10.3389/fpls.2024.1397632 (PMC11608969; doi:10.3389/fpls.2024.1397632)
Supplement: Supplementary Methods 2 — Amplicon sequencing method and data analysis. [file DataSheet5.pdf]

MATERIALS AND METHODS

Plant Material

All transfection experiments were performed using DH12075 seeds. *In vitro* propagation, protoplast isolation, transfection and regeneration were performed as described in Sahab et al. (2019) with some modifications.

Seed Germination

Seeds were surface sterilized using surface-sterilizing agents (70% (v/v) ethanol for 2 min and 20% (v/v) commercial "Domestos" bleach solution (12.5 g/L active chlorine) with 0.1% (v/v) Tween 20) for 20 min, and then rinsed thoroughly with sterile water. Surface sterilized seeds were planted on germination medium (Half strength Murashige & Skoog (MS)/B5 vitamins, 1% sucrose, 0.8% agar; pH 5.8).

Protoplast Isolation

Mesophyll-derived protoplasts were isolated from sterile shoot cultures of *Brassica napus* (DH10275). Two to three fully expanded leaves were taken from 3- to 4-week-old shoot cultures and placed in a 100 X 20 mm plastic Petri dish. The leaf tissue was moistened using 1–2 mL of digestion buffer and cut into thin 0.5–1 mm strips with a sharp scalpel. Then, the leaf strips were transferred to a fresh 100 X 20 mm Petri dish containing 30 mL of digestion buffer (1.5% (w/v) cellulase Onozuka^{TM} R-10 (Yakult Pharmaceutical Co., LTD., Tokyo, Japan), 0.6% (w/v) Macerozyme^{TM} R-10 (Yakult Pharmaceutical Co., Ltd.), 0.4M mannitol, 10mM MES at pH 5.7). The isolated protoplasts were passed through a 70µm nylon cell strainer into a 50 ml Falcon tube. The filtered protoplast suspension was centrifuged at 70 g for 10 min. The pellet was resuspended using 15 ml of W5/wash buffer (Menczel et al., 1981) and centrifuged at 70 g for 10 min, and this process was repeated twice. Finally, the Pellet was re-suspended in 5 ml W5 buffer. Protoplast yield was assessed using a hemocytometer, and viability was tested by staining using Evans blue. Protoplast density was adjusted to 1X 10^6 protoplasts per ml using W5 buffer.

Sodium Alginate based bead-type culture and microcalli formation

The transfected protoplasts were centrifuged at 70 g using a swing-out rotor for 10 minutes, and the supernatant was carefully removed. The protoplasts were then resuspended in 5.0 mL of 0.5 M mannitol, and the wash was repeated by centrifugation at 70 g for another 10 minutes. After removing the supernatant, the protoplast pellet was resuspended in 1.0 mL of 0.5 M mannitol. The protoplast suspension was incubated on ice until needed. An equal volume of 1.0% sodium alginate, prepared

using 0.4 M Mannitol, was added to the suspended protoplasts and mixed gently. The protoplast suspension mix was placed on ice until it was embedded. Next, 4 mL of bead-forming solution was added into a sterile six-well plate. The protoplast suspension was aspirated using a 1 mL pipette and added dropwise to the bead-forming solution (0.4 M mannitol, 50 mM CaCl2; pH 5.8) within the plate. The protoplast suspension was incubated for 20–30 minutes at room temperature until sodium alginate beads formed. The bead-forming solution was carefully removed and replaced with 4 mL of a 1:2 mixture of K3 + H:A media (Spangenberg and Potrykus, 1996). The protoplasts were incubated for 3–4 weeks in the dark at 22 °C in an incubator shaker set to 50 rpm. Protoplast division and the absence of any contaminations were observed under an inverted microscope. After 3–4 weeks, the microcalli (0.5–1.0 mm-sized calli) were released by incubating the sodium alginate beads with 3–4 mL of depolymerization buffer (0.3 M mannitol, 20 mM sodium citrate; pH 5.8).

Callus proliferation and shoot regeneration

The microcalli were resuspended in 5 mL of liquid A media (Spangenberg and Potrykus, 1996), and gently mixed using a sterile 10 mL disposable pipette. 1 mL of the resuspended microcalli was transferred per callus proliferation medium plate (MS/MS vitamins, 3.5% sucrose, 500 mg/L MES, 5 µM BAP, 5 µM NAA, 5 µm 2, 4-D, 0.7% agarose type I; pH 6.0 with 30 mL of media poured into each 100 X 20 mm sterile Petri dish). The cultures were maintained at 22 °C in 16 h/d light (30 µmol m^{-2} s^{-1}). Green-coloured microcalli were transferred to shoot regeneration medium (MS/MS vitamins, 3.0% sucrose, 500 mg/L MES, 5 mg/L 2i P, 0.5 µM NAA, 0.7% agarose type I; pH 5.8 with 30 mL of media poured in each 100 X 20 mm sterile Petri dish). All green calli that had produced shoot primordia were transferred to shoot elongation medium (MS/B5 vitamins, 2% sucrose, 500 mg/L MES, 2 µM BAP, 0.1 µM GA-3, 0.8% agar; pH 5.8, with 50 mL of media poured into each STERICON™ 8 sterilized culture vessel). The shoot cultures were maintained in SEM for 2 weeks. Healthy shoots that survived and grew normally were transferred to root induction media (Half strength MS/B5 vitamins, 1% sucrose, 500 mg/L MES, 2.5 µM IBA, 0.6% agar; pH 5.8 with 100 mL of media poured in STERICON™ 13 sterilized culture vessel). All *in vitro* cultures in this study were maintained in a controlled climate chamber with a temperature of 23 ◦C/18 ◦C (day/night) and a 16 h photoperiod with a light intensity of 40 µmol m−2 s−1. After successful root initiation, rooted plantlets were transferred to soil (Figure. 1C).

References:

Menczel, L., Nagy, F., Kiss, Z. R., & Maliga, P. (1981). Streptomycin resistant and sensitive somatic hybrids of Nicotiana tabacum + Nicotiana knightiana: correlation of resistance to N. tabacum plastids. *TAG. Theoretical and applied genetics. Theoretische und angewandte Genetik*, *59*(3), 191–195. https://doi.org/10.1007/BF00264975

Sahab, S., Hayden, M. J., Mason, J., & Spangenberg, G. (2019). Mesophyll Protoplasts and PEG-Mediated Transfections: Transient Assays and Generation of Stable Transgenic Canola Plants. *Methods in molecular biology (Clifton, N.J.)*, *1864*, 131–152. https://doi.org/10.1007/978-1-4939-8778-8_10

Spangenberg G, Potrykus I (1996) In: Potrykus I, Spangenberg G (eds) Polyethylene glycol-mediated direct gene transfer to tobacco protoplasts and regeneration of transgenic plants: gene transfer to plants. Springer-Verlag, Berlin, Heidelberg, New York, pp 59–65
