# Supplementary material for: Efficient multi-allelic genome editing via CRISPR–Cas9 ribonucleoprotein-based delivery to Brassica napus mesophyll protoplasts
Source: Front Plant Sci. 2024 Nov 18;15:1397632. doi: 10.3389/fpls.2024.1397632 (PMC11608969; doi:10.3389/fpls.2024.1397632)
Supplement: Supplementary Table 2 — ddPCR probes and primers used to quantify the frequency of mutations in CENH3 alleles. [file DataSheet3.pdf]

Supplementary Table 1. Details of a single guide RNA targeting the 5'UTR of the CENH3 gene

CRISPR RNA Name	Single Guide RNA Sequence
R026	$^{m}C^{m}A^{m}$ACUCAUAAGAGGCAUGAAGUUUUAGAGCUAGAAAUAGCAAGUUAAAAUAAGGCUAGUCCGUUUCAACUUGAAAAAGUGGCACCGAGUCGGUGC$^{m}U^{m}U^{m}$UU

spacer sequence is underlined. O-Methyl RNA bases are indicated in superscript

Supplementary Table 2. ddPCR probes and primers used to quantify the frequency of mutation of *CENH3* alleles

Name	Type	Nucleotide sequence (5’-3')	Tm (°C)
Bn_R026_R035_F072_f2	Primer	ATGTGCTGAGCAAACCCTCT	63.4
Bn_R026_R035_F072_r1	Primer	AGATTATTTGTTTTCCCGCTTCTCC	63
Bn_R026_R035_abl_FAM	Probe[1]	*FAM*-ACCATTCATGCCTCTTATGAGTTGTAGTCTG-*BHQ1*	67
Bn_F072_HEX	Probe[2]	*HEX*-TCCTTCTGCTTTCTTTCACACCAACGC-*BHQ1*	68.3

[1]ablation probe; [2]control probe
