# Supplementary material for: Efficient multi-allelic genome editing via CRISPR–Cas9 ribonucleoprotein-based delivery to Brassica napus mesophyll protoplasts
Source: Front Plant Sci. 2024 Nov 18;15:1397632. doi: 10.3389/fpls.2024.1397632 (PMC11608969; doi:10.3389/fpls.2024.1397632)
Supplement: Supplementary Table 4 — Raw results of droplet digital PCR ablation assay for canola shoot samples derived from protoplasts transfected with a CRISPR-CAS9 RNP targeting BnCENH3 homoeologs. [file DataSheet2.pdf]

Plant ID	ddPCR CENH3 ablate frequency (%)	No. of putative ablated alleles
BNICBCAS9_1	79	3
BNICBCAS9_2	100	4
BNICBCAS9_3	2	0
BNICBCAS9_4	100	4
BNICBCAS9_5	1	0
BNICBCAS9_6	60	2
BNICBCAS9_7	100	4
BNICBCAS9_8	1	0
BNICBCAS9_9	100	4
BNICBCAS9_10	1	0
BNICBCAS9_11	100	4
BNICBCAS9_12	2	0
BNICBCAS9_13	100	4
BNICBCAS9_14	78	3
BNICBCAS9_15	56	2
BNICBCAS9_16	2	0
BNICBCAS9_17	100	4
BNICBCAS9_18	100	4
BNICBCAS9_19	100	4
BNICBCAS9_20	52	2
BNICBCAS9_21	55	2
BNICBCAS9_22	100	4
BNICBCAS9_23	100	4
BNICBCAS9_24	100	4
BNICBCAS9_25	28	1
BNICBCAS9_26	100	4
BNICBCAS9_27	100	4
BNICBCAS9_28	82	3
BNICBCAS9_29	81	3
BNICBCAS9_30	100	4
BNICBCAS9_31	100	4
BNICBCAS9_32	100	4
BNICBCAS9_33	80	3
BNICBCAS9_34	29	1
BNICBCAS9_35	1	0
BNICBCAS9_36	0	0
BNICBCAS9_37	1	0
BNICBCAS9_38	2	0
BNICBCAS9_39	32	1
BNICBCAS9_40	1	0
BNICBCAS9_41	0	0
BNICBCAS9_42	85	3
BNICBCAS9_43	0	0
BNICBCAS9_44	28	1

Plant ID	ddPCR CENH3 ablate frequency (%)	No. of putative ablated alleles
BNICBCAS9_45	50	2
BNICBCAS9_46	100	4
BNICBCAS9_47	100	4
BNICBCAS9_48	100	4
BNICBCAS9_49	51	2
BNICBCAS9_50	0	0
BNICBCAS9_51	100	4
BNICBCAS9_52	0	0
BNICBCAS9_53	0	0
BNICBCAS9_54	100	4
BNICBCAS9_55	100	4
BNICBCAS9_56	28	1
BNICBCAS9_57	0	0
BNICBCAS9_58	100	4
BNICBCAS9_59	0	0
BNICBCAS9_60	0	0
BNICBCAS9_61	100	4
BNICBCAS9_62	100	4
BNICBCAS9_63	1	0
BNICBCAS9_64	100	4
BNICBCAS9_65	100	4
BNICBCAS9_66	0	0
BNICBCAS9_67	0	0
BNICBCAS9_68	83	3
BNICBCAS9_69	100	4
BNICBCAS9_70	100	4
BNICBCAS9_71	100	4
BNICBCAS9_72	0	0
BNICBCAS9_73	0	0
BNICBCAS9_74	100	4
BNICBCAS9_75	81	3
BNICBCAS9_76	100	4
BNICBCAS9_77	100	4
BNICBCAS9_78	0	0
BNICBCAS9_79	100	4
BNICBCAS9_80	98	4
BNICBCAS9_81	100	4
BNICBCAS9_82	83	3
BNICBCAS9_83	87	3
BNICBCAS9_84	51	2
BNICBCAS9_85	0	0
BNICBCAS9_86	0	0
BNICBCAS9_87	78	3
BNICBCAS9_88	100	4

Plant ID	ddPCR CENH3 ablate frequency (%)	No. of putative ablated alleles
BNICBCAS9_89	1	0
BNICBCAS9_90	100	4
BNICBCAS9_91	2	0
BNICBCAS9_92	59	2
BNICBCAS9_93	100	4
BNICBCAS9_94	2	0
BNICBCAS9_95	100	4
BNICBCAS9_96	3	0
BNICBCAS9_97	100	4
BNICBCAS9_98	100	4
BNICBCAS9_99	100	4
BNICBCAS9_100	81	3
BNICBCAS9_101	2	0
BNICBCAS9_102	100	4
BNICBCAS9_103	4	0
BNICBCAS9_104	100	4
BNICBCAS9_105	100	4
BNICBCAS9_106	100	4
BNICBCAS9_107	100	4
BNICBCAS9_108	1	0
BNICBCAS9_109	33	1
BNICBCAS9_110	100	4
BNICBCAS9_111	100	4
BNICBCAS9_112	33	1
BNICBCAS9_113	89	3
BNICBCAS9_114	1	0
BNICBCAS9_115	82	3
BNICBCAS9_116	100	4
BNICBCAS9_117	100	4
BNICBCAS9_118	1	0
BNICBCAS9_119	100	4
BNICBCAS9_120	1	0
BNICBCAS9_121	79	3
BNICBCAS9_122	0	0
BNICBCAS9_123	43	2
BNICBCAS9_124	1	0
BNICBCAS9_125	100	4
BNICBCAS9_126	100	4
BNICBCAS9_127	100	4
BNICBCAS9_128	100	4
BNICBCAS9_129	100	4
BNICBCAS9_130	100	4
BNICBCAS9_131	0	0
BNICBCAS9_132	91	4

Plant ID	ddPCR CENH3 ablate frequency (%)	No. of putative ablated alleles
BNICBCAS9_133	1	0
BNICBCAS9_134	1	0
BNICBCAS9_135	100	4
BNICBCAS9_136	27	1
BNICBCAS9_137	100	4
BNICBCAS9_138	79	3
BNICBCAS9_139	100	4
BNICBCAS9_140	1	0
BNICBCAS9_141	100	4
BNICBCAS9_142	1	0
BNICBCAS9_143	100	4
BNICBCAS9_144	100	4
BNICBCAS9_145	0	0
BNICBCAS9_146	100	4
BNICBCAS9_147	1	0
BNICBCAS9_148	0	0
BNICBCAS9_149	0	0
BNICBCAS9_150	0	0
BNICBCAS9_151	1	0
BNICBCAS9_152	0	0
BNICBCAS9_153	100	4
BNICBCAS9_154	100	4
BNICBCAS9_155	0	0
BNICBCAS9_156	100	4
BNICBCAS9_157	100	4
BNICBCAS9_158	0	0
BNICBCAS9_159	81	3
BNICBCAS9_160	100	4
BNICBCAS9_161	100	4
BNICBCAS9_162	100	4
BNICBCAS9_163	1	0
BNICBCAS9_164	100	4
BNICBCAS9_165	39	1
BNICBCAS9_166	0	0
BNICBCAS9_167	100	4
BNICBCAS9_168	80	3
BNICBCAS9_169	1	0
BNICBCAS9_170	100	4
BNICBCAS9_171	76	3
BNICBCAS9_172	28	1
BNICBCAS9_173	100	4
BNICBCAS9_174	100	4
BNICBCAS9_175	100	4
BNICBCAS9_176	100	4

Plant ID	ddPCR CENH3 ablate frequency (%)	No. of putative ablated alleles
BNICBCAS9_177	100	4
BNICBCAS9_178	100	4
BNICBCAS9_179	3	0
BNICBCAS9_180	100	4
BNICBCAS9_181	1	0
BNICBCAS9_182	2	0
BNICBCAS9_183	2	0
BNICBCAS9_184	100	4
BNICBCAS9_185	100	4
BNICBCAS9_186	100	4
BNICBCAS9_187	100	4
BNICBCAS9_188	100	4
BNICBCAS9_189	2	0
BNICBCAS9_190	100	4
BNICBCAS9_191	100	4
BNICBCAS9_192	2	0
BNICBCAS9_193	100	4
BNICBCAS9_194	100	4
BNICBCAS9_195	1	0
BNICBCAS9_196	80	3
BNICBCAS9_197	100	4
BNICBCAS9_198	2	0
BNICBCAS9_199	2	0
BNICBCAS9_200	3	0
BNICBCAS9_201	100	4
BNICBCAS9_202	100	4
BNICBCAS9_203	100	4
BNICBCAS9_204	2	0
BNICBCAS9_205	52	2
BNICBCAS9_206	2	0
BNICBCAS9_207	100	4
BNICBCAS9_208	100	4
BNICBCAS9_209	100	4
BNICBCAS9_210	29	1
BNICBCAS9_211	1	0
BNICBCAS9_212	100	4
BNICBCAS9_213	100	4
BNICBCAS9_214	0	0
BNICBCAS9_215	1	0
BNICBCAS9_216	100	4
BNICBCAS9_217	1	0
BNICBCAS9_218	86	3
BNICBCAS9_219	100	4
BNICBCAS9_220	54	2

Plant ID	ddPCR CENH3 ablate frequency (%)	No. of putative ablated alleles
BNICBCAS9_221	100	4
BNICBCAS9_222	100	4
BNICBCAS9_223	100	4
BNICBCAS9_224	55	2
BNICBCAS9_225	100	4
BNICBCAS9_226	100	4
BNICBCAS9_227	84	3
BNICBCAS9_228	29	1
BNICBCAS9_229	2	0
BNICBCAS9_230	99	4
BNICBCAS9_231	2	0
BNICBCAS9_232	100	4
BNICBCAS9_233	1	0
BNICBCAS9_234	2	0
BNICBCAS9_235	85	3
BNICBCAS9_236	0	0
BNICBCAS9_237	100	4
BNICBCAS9_238	100	4
BNICBCAS9_239	100	4
BNICBCAS9_240	100	4
BNICBCAS9_241	100	4
BNICBCAS9_242	58	2
BNICBCAS9_243	100	4
BNICBCAS9_244	52	2
BNICBCAS9_245	1	0
BNICBCAS9_246	79	3
BNICBCAS9_247	100	4
BNICBCAS9_248	26	1
BNICBCAS9_249	28	1
BNICBCAS9_250	58	2
BNICBCAS9_251	2	0
BNICBCAS9_252	1	0
BNICBCAS9_253	77	3
BNICBCAS9_254	82	3
BNICBCAS9_255	3	0
BNICBCAS9_256	100	4
BNICBCAS9_257	100	4
BNICBCAS9_258	0	0
BNICBCAS9_259	0	0
BNICBCAS9_260	0	0
BNICBCAS9_261	100	4
BNICBCAS9_262	100	4
BNICBCAS9_263	100	4
BNICBCAS9_264	100	4

Plant ID	ddPCR CENH3 ablate frequency (%)	No. of putative ablated alleles
BNICBCAS9_265	100	4
BNICBCAS9_266	100	4
BNICBCAS9_267	2	0
BNICBCAS9_268	100	4
BNICBCAS9_269	1	0
BNICBCAS9_270	100	4
BNICBCAS9_271	2	0
BNICBCAS9_272	2	0
BNICBCAS9_273	2	0
BNICBCAS9_274	100	4
BNICBCAS9_275	2	0
BNICBCAS9_276	99	4
BNICBCAS9_277	100	4
BNICBCAS9_278	53	2
BNICBCAS9_279	82	3
BNICBCAS9_280	54	2
BNICBCAS9_281	1	0
BNICBCAS9_282	1	0
BNICBCAS9_283	100	4
BNICBCAS9_284	100	4
BNICBCAS9_285	1	0
BNICBCAS9_286	100	4
BNICBCAS9_287	100	4
BNICBCAS9_288	30	1
BNICBCAS9_289	100	4
BNICBCAS9_290	100	4
BNICBCAS9_291	2	0
BNICBCAS9_292	1	0
BNICBCAS9_293	100	4
BNICBCAS9_294	85	3
BNICBCAS9_295	100	4
BNICBCAS9_296	1	0
BNICBCAS9_297	1	0
BNICBCAS9_298	100	4
BNICBCAS9_299	100	4
BNICBCAS9_300	100	4
BNICBCAS9_301	85	3
BNICBCAS9_302	100	4
BNICBCAS9_303	100	4
BNICBCAS9_304	1	0
BNICBCAS9_305	0	0
BNICBCAS9_306	0	0
BNICBCAS9_307	0	0
BNICBCAS9_308	100	4

Plant ID	ddPCR CENH3 ablate frequency (%)	No. of putative ablated alleles
BNICBCAS9_309	77	3
BNICBCAS9_310	100	4
BNICBCAS9_311	100	4
BNICBCAS9_312	56	2
BNICBCAS9_313	30	1
BNICBCAS9_314	0	0
BNICBCAS9_315	100	4
BNICBCAS9_316	100	4
BNICBCAS9_317	100	4
BNICBCAS9_318	100	4
BNICBCAS9_319	1	0
BNICBCAS9_320	100	4
BNICBCAS9_321	1	0
BNICBCAS9_322	100	4
BNICBCAS9_323	79	3
BNICBCAS9_324	100	4
BNICBCAS9_325	80	3
BNICBCAS9_326	100	4
BNICBCAS9_327	99	4
BNICBCAS9_328	100	4
BNICBCAS9_329	1	0
BNICBCAS9_330	100	4
BNICBCAS9_331	0	0
BNICBCAS9_332	100	4
BNICBCAS9_333	100	4
BNICBCAS9_334	100	4
BNICBCAS9_335	1	0
BNICBCAS9_336	0	0
BNICBCAS9_337	1	0
BNICBCAS9_338	0	0
BNICBCAS9_339	77	3
BNICBCAS9_340	100	4
BNICBCAS9_341	0	0
BNICBCAS9_342	78	3
BNICBCAS9_343	79	3
BNICBCAS9_344	100	4
BNICBCAS9_345	100	4
BNICBCAS9_346	100	4
BNICBCAS9_347	100	4
BNICBCAS9_348	100	4
BNICBCAS9_349	100	4
BNICBCAS9_350	0	0
BNICBCAS9_351	100	4
BNICBCAS9_352	100	4

Plant ID	ddPCR CENH3 ablate frequency (%)	No. of putative ablated alleles
BNICBCAS9_353	3	0
BNICBCAS9_354	3	0
BNICBCAS9_355	100	4
BNICBCAS9_356	52	2
BNICBCAS9_357	2	0
BNICBCAS9_358	99	4
BNICBCAS9_359	2	0
BNICBCAS9_360	97	4
BNICBCAS9_361	100	4
BNICBCAS9_362	100	4
BNICBCAS9_363	2	0
BNICBCAS9_364	2	0
BNICBCAS9_365	1	0
BNICBCAS9_366	29	1
BNICBCAS9_367	1	0
BNICBCAS9_368	100	4
BNICBCAS9_369	100	4
BNICBCAS9_370	100	4
BNICBCAS9_371	100	4
BNICBCAS9_372	100	4
BNICBCAS9_373	1	0
BNICBCAS9_374	100	4
BNICBCAS9_375	100	4
BNICBCAS9_376	100	4
BNICBCAS9_377	100	4
BNICBCAS9_378	100	4
BNICBCAS9_379	2	0
BNICBCAS9_380	100	4
BNICBCAS9_381	99	4
BNICBCAS9_382	2	0
BNICBCAS9_383	99	4
BNICBCAS9_384	1	0
BNICBCAS9_385	99	4
BNICBCAS9_386	27	1
BNICBCAS9_387	27	1
BNICBCAS9_388	70	3
BNICBCAS9_389	100	4
BNICBCAS9_390	100	4
BNICBCAS9_391	1	0
BNICBCAS9_392	1	0
BNICBCAS9_393	99	4
BNICBCAS9_394	100	4
BNICBCAS9_395	87	3
BNICBCAS9_396	1	0

Plant ID	ddPCR CENH3 ablate frequency (%)	No. of putative ablated alleles
BNICBCAS9_397	100	4
BNICBCAS9_398	1	0
BNICBCAS9_399	100	4
BNICBCAS9_400	100	4
BNICBCAS9_401	1	0
BNICBCAS9_402	0	0
BNICBCAS9_403	100	4
BNICBCAS9_404	22	1
BNICBCAS9_405	1	0
BNICBCAS9_406	3	0
BNICBCAS9_407	53	2
BNICBCAS9_408	54	2
BNICBCAS9_409	100	4
BNICBCAS9_410	100	4
BNICBCAS9_411	1	0
BNICBCAS9_412	100	4
BNICBCAS9_413	82	3
BNICBCAS9_414	100	4
BNICBCAS9_415	100	4
BNICBCAS9_416	100	4
BNICBCAS9_417	82	3
BNICBCAS9_418	100	4
BNICBCAS9_419	100	4
BNICBCAS9_420	1	0
BNICBCAS9_421	31	1
BNICBCAS9_422	100	4
BNICBCAS9_423	100	4
BNICBCAS9_424	1	0
BNICBCAS9_425	100	4
BNICBCAS9_426	79	3
BNICBCAS9_427	1	0
BNICBCAS9_428	1	0
BNICBCAS9_429	1	0
BNICBCAS9_430	100	4
BNICBCAS9_431	100	4
BNICBCAS9_432	100	4
BNICBCAS9_433	1	0
BNICBCAS9_434	99	4
BNICBCAS9_435	1	0
BNICBCAS9_436	93	4
BNICBCAS9_437	100	4
BNICBCAS9_438	100	4
BNICBCAS9_439	1	0
BNICBCAS9_440	3	0

Plant ID	ddPCR CENH3 ablate frequency (%)	No. of putative ablated alleles
BNICBCAS9_441	100	4
BNICBCAS9_442	99	4
BNICBCAS9_443	11	0
BNICBCAS9_444	99	4
BNICBCAS9_445	99	4
BNICBCAS9_446	57	2
BNICBCAS9_447	4	0
BNICBCAS9_448	4	0
BNICBCAS9_449	100	4
BNICBCAS9_450	37	1
BNICBCAS9_451	99	4
BNICBCAS9_452	99	4
BNICBCAS9_453	15	0
BNICBCAS9_454	98	4
BNICBCAS9_455	100	4
BNICBCAS9_456	100	4
BNICBCAS9_457	57	2
BNICBCAS9_458	100	4
BNICBCAS9_459	100	4
BNICBCAS9_460	11	0
BNICBCAS9_461	99	4
BNICBCAS9_462	100	4
BNICBCAS9_463	100	4
BNICBCAS9_464	100	4
BNICBCAS9_465	100	4
BNICBCAS9_466	100	4
BNICBCAS9_467	8	0
BNICBCAS9_468	8	0
BNICBCAS9_469	9	0
BNICBCAS9_470	8	0
BNICBCAS9_471	100	4
BNICBCAS9_472	100	4
BNICBCAS9_473	4	0
BNICBCAS9_474	5	0
BNICBCAS9_475	99	4
BNICBCAS9_476	6	0
BNICBCAS9_477	100	4
BNICBCAS9_478	100	4
BNICBCAS9_479	100	4
BNICBCAS9_480	6	0
BNICBCAS9_481	100	4
BNICBCAS9_482	100	4
BNICBCAS9_483	100	4
BNICBCAS9_484	99	4

Plant ID	ddPCR CENH3 ablate frequency (%)	No. of putative ablated alleles
**BNICBCAS9_485**	59	2
**BNICBCAS9_486**	5	0
**BNICBCAS9_487**	100	4
**BNICBCAS9_488**	100	4
**BNICBCAS9_489**	56	2
**BNICBCAS9_490**	3	0
**BNICBCAS9_491**	100	4
**BNICBCAS9_492**	6	0
**BNICBCAS9_493**	99	4
**BNICBCAS9_494**	63	2
**BNICBCAS9_495**	84	3
**BNICBCAS9_496**	31	1
**BNICBCAS9_497**	86	3
**BNICBCAS9_498**	5	0
**BNICBCAS9_499**	100	4
**BNICBCAS9_500**	3	0
**BNICBCAS9_501**	100	4
**BNICBCAS9_502**	6	0
**BNICBCAS9_503**	5	0
**BNICBCAS9_504**	100	4
**BNICBCAS9_505**	4	0
**BNICBCAS9_506**	66	2
**BNICBCAS9_507**	100	4
**BNICBCAS9_508**	57	2
**BNICBCAS9_509**	99	4
**BNICBCAS9_510**	100	4
**BNICBCAS9_511**	100	4
**BNICBCAS9_512**	100	4
**BNICBCAS9_513**	100	4
**BNICBCAS9_514**	82	3
**BNICBCAS9_515**	4	0
**BNICBCAS9_516**	2	0
**BNICBCAS9_517**	56	2
**BNICBCAS9_518**	55	2
**BNICBCAS9_519**	4	0
**BNICBCAS9_520**	100	4
**BNICBCAS9_521**	33	1
**BNICBCAS9_522**	26	1
**BNICBCAS9_523**	100	4
**BNICBCAS9_524**	100	4
**BNICBCAS9_525**	5	0
